# Nursing home characteristics associated with methicillin-resistant *Staphylococcus aureus* (MRSA) Burden and Transmission

**DOI:** 10.1186/1471-2334-12-269

**Published:** 2012-10-24

**Authors:** Courtney R Murphy, Victor Quan, Diane Kim, Ellena Peterson, Matthew Whealon, Grace Tan, Kaye Evans, Hildy Meyers, Michele Cheung, Bruce Y Lee, Dana B Mukamel, Susan S Huang

**Affiliations:** 1 Division of Infectious Diseases and Health Policy Research Institute, University of California Irvine School of Medicine, Irvine, USA; 2 Department of Pathology and Laboratory Medicine, University of California Irvine School of Medicine, Irvine, USA; 3 Epidemiology and Assessment Program, Orange County Health Care Agency, Santa Ana, USA; 4 Department of Medicine, Epidemiology and Biomedical Informatics, University of Pittsburgh School of Medicine and Graduate School of Public Health, Pittsburgh, USA; 5 Department of Medicine and Health Policy Research Institute, University of California Irvine School of Medicine, Irvine, USA

**Keywords:** MRSA, Healthcare-associated infection, Long-term care, Nursing home

## Abstract

**Background:**

MRSA prevalence in nursing homes often exceeds that in hospitals, but reasons for this are not well understood. We sought to measure MRSA burden in a large number of nursing homes and identify facility characteristics associated with high MRSA burden.

**Methods:**

We performed nasal swabs of residents from 26 nursing homes to measure MRSA importation and point prevalence, and estimate transmission. Using nursing home administrative data, we identified facility characteristics associated with MRSA point prevalence and estimated transmission risk in multivariate models.

**Results:**

We obtained 1,649 admission and 2,111 point prevalence swabs. Mean MRSA point prevalence was 24%, significantly higher than mean MRSA admission prevalence, 16%, (paired t-test, p<0.001), with a mean estimated MRSA transmission risk of 16%.

In multivariate models, higher MRSA point prevalence was associated with higher admission prevalence (p=0.005) and higher proportions of residents with indwelling devices (p=0.01). Higher estimated MRSA transmission risk was associated with higher proportions of residents with diabetes (p=0.01) and lower levels of social engagement (p=0.03).

**Conclusions:**

MRSA importation was a strong predictor of MRSA prevalence, but MRSA burden and transmission were also associated with nursing homes caring for more residents with chronic illnesses or indwelling devices. Frequent social interaction among residents appeared to be protective of MRSA transmission, suggesting that residents healthy enough to engage in group activities do not incur substantial risks of MRSA from social contact. Identifying characteristics of nursing homes at risk for high MRSA burden and transmission may allow facilities to tailor infection control policies and interventions to mitigate MRSA spread.

## Background

The burden of methicillin-resistant *Staphylococcus aureus* (MRSA) in nursing homes is less well studied compared to hospital settings. Nevertheless, MRSA carriage in nursing homes can reach or exceed 50% of residents [1-8], often surpassing that found in general hospital wards (6-12%) [9-11] and in intensive care units (7-24%) [12-16].

Predictors of high MRSA burden in nursing homes are not known, but are likely to include factors that predict acquisition in other healthcare settings, such as diabetes, skin breaks, dialysis, and the presence of indwelling devices [3,17-19]. In addition, nursing homes may have unique risk factors vs. hospitals. Social interaction between residents distinguishes nursing homes from acute care settings, and has an unknown impact upon MRSA acquisition. In turn, factors that influence a resident’s ability to socialize—including acuity level and mobility—may influence their risk of acquiring MRSA. Furthermore, facility-level characteristics may influence individual risk, especially for contagious pathogens such as MRSA [20,21]. Residing in a nursing home where most residents are at high risk for MRSA may increase the likelihood of acquisition even for residents who lack these specific risk factors. Elucidating facility characteristics associated with high MRSA burden may be helpful in identifying nursing homes that would benefit from intervention.

We sought to measure MRSA burden in a large number of nursing homes within a single metropolitan county and to identify facility characteristics associated with elevated MRSA burden and transmission.

## Methods

### Screening nursing home residents for MRSA

We measured MRSA carriage among nursing home residents in a convenience sample of 26 of the 72 nursing homes in Orange County, California from October 2008-May 2011. In each nursing home, a point prevalence screening was performed of the bilateral nares of up to 100 residents. In addition, we performed an admission prevalence screening of up to 100 consecutive residents within 3 days of admission. For nursing homes with low bed turnover, a lesser number of residents were screened (30–50), and for nursing homes with an average length of stay in years, admission screening was not performed. For each swabbed resident, we recorded the nursing home day of swab collection, whether there was a known history of MRSA, and whether the resident shared a room.

Bilateral nares swabs (BBL Culture Swabs, Sparks, Maryland) were transported to a central microbiology laboratory and plated within 12 hours. Samples were cultured onto 5% sheep blood agar (BBL) and selective and differential chromogenic media for MRSA, Spectra MRSA (Remel, Lenexa, Kansas) and incubated for 24 hours. MRSA was identified using Spectra MRSA and confirmed agglutination testing and Gram stain. The Institutional Review Board of the University of California Regents approved this study. This study was completed as a quality improvement project through participating nursing homes and consequently explicit consent was not obtained. However, residents were able to refuse to participate.

### Nursing home variables

Nursing home characteristics were obtained from the Minimum Data Set (MDS), version 2.0 [22]. MDS is an individual resident-level dataset containing assessments of physical, psychological and psycho-social functioning mandated for all residents of Medicare and Medicaid licensed nursing homes in the United States. While we did not obtain data from direct interviews with patients, MDS data has been extensively used in long-term care research and has been validated to measure outcomes such as depression, aggressive behavior and social engagement [23-25]. We calculated the proportion of nursing home residents with various MDS characteristics based upon 2009 data, which represented the most recently available data at the time of our analysis. Variables included facility characteristics such as volume and turnover, as well as the percent of residents with various demographics, comorbidities, and social engagement scores. We used a previously validated social engagement score calculated from MDS data that measures how readily residents interact with others and how willing they are to initiate and participate in activities [25]. The average number of daily direct care hours for nursing staff, average resource utilization group scores (RUGS), and average activities of daily living scores (ADLS) were obtained from Long Term Care Focus [26] for 2007, the most recent year available. RUGS is a facility-level score that reflects the average level of care required by residents, based upon residents’ comorbidities, dependence upon caregivers, and required amount of physical and occupation therapy. The RUGS, ADLS and social engagement scores were used as measures of resident functional status.

### Analysis

Admission prevalence was calculated as the percent of residents swabbed upon nursing home admission that were found to be MRSA carriers. Point prevalence was calculated as the percent of patients found to be MRSA carriers when swabbing a representative sample of nursing home residents on a given day. We performed paired t-tests comparing MRSA admission vs. point prevalence and compared nursing home length-of-stay (LOS) for MRSA-positive vs. MRSA-negative residents, since higher LOS for MRSA-positive residents could contribute to higher MRSA prevalence over time. Finally, for nursing homes with both MRSA admission and point prevalence measures, we calculated an estimated facility-specific transmission risk based upon the difference in percentages between admission and point prevalence divided by the percent of residents that were admitted without MRSA.

We tested associations of facility-level characteristics with nursing home MRSA point prevalence and estimated transmission risk. Variables with p<0.1 on bivariate testing using linear regression models were entered into a multivariate facility-level linear regression model and retained at alpha = 0.05. A maximum of two facility-level variables per outcome were permitted in each multivariate model to prevent overfitting of the model due to sample size (26 total facilities). All variables and outcomes were continuous, except for MRSA admission prevalence and RUGS, which were dichotomized into high vs. low groups based upon median values.

## Results

We obtained 1,649 admission and 2,111 point prevalence swabs from 26 Orange County nursing homes. Of admission swabs, 269 were MRSA positive, while 571 point prevalence swabs were MRSA positive. We were unable to obtain admission prevalence swabs from 7 facilities, due to small facility size and minimal resident turnover. Less than 5% of residents refused to be swabbed. Facility characteristics are listed in Table 1. Across all nursing homes, mean MRSA admission prevalence was 16% (standard deviation (SD) 8), while mean MRSA point prevalence was 24% (SD 13) (Table 2). Overall, mean MRSA point prevalence was significantly higher than mean MRSA admission prevalence (paired t-test, p<0.001) despite similar nursing home length-of-stay among MRSA-positive and MRSA-negative residents (399 vs. 436 days; t-test, p=0.26). Although overall MRSA point prevalence was significantly higher than admission prevalence, these measures were highly correlated (Pearson coefficient =0.77). Nevertheless, it was noteworthy that some facilities had nearly equal admission prevalences, but substantially different point prevalences (Table 2). For example, nursing homes 5 and 6 admit 10% and 11% MRSA carriers, respectively. However, MRSA point prevalence is 25% at nursing home 5, while point prevalence remains stable in nursing home 6, at 7%. In bivariate models (Table 3), MRSA point prevalence was associated (p<0.1) with annual admissions, MRSA admission prevalence, the percent of residents admitted from acute care hospitals, and the percent of residents with select comorbidities (diabetes, indwelling devices, skin lesions, fecal incontinence). Results for continuous variables are reported per 10% increase; for example, a 10% absolute increase in the percent of residents with diabetes was associated with a 7.2% absolute increase in MRSA point prevalence.

**Table 1:** Characteristics of 26 Nursing Homes in Orange County, California

**Nursing Home Characteristic**	**Median (Range)**	**Mean (Standard Deviation)**
Number of Beds	99 (24 – 255)	110 (58)
Median Length of Stay (days)	102 (25 – 753)	149 (189)
Annual Admissions	262 (18 – 1526)	421 (425)
% Annual Resident Turnover	15% (1 – 70)	19% (14)
Average Direct Care Nursing Staff Hours (per resident day)	3.5 (1.9 – 7.8)	3.9 (1.4)
***Demographics****(as*% *of all facility residents)*		
Age		
% <65 years old	28 (0 – 86)	30 (28)
% 65- <85 years old	44 (9 – 57)	40 (13)
% >85 years old	25 (2 – 72)	30 (20)
% Male	43 (21 – 67)	42 (12)
Race and Ethnicity		
% White	84 (12 – 99)	81 (17)
% Black	1 (0 – 9)	3 (3)
% American Indian/Alaskan Native	0 (0 – 2)	0 (1)
% Asian/Pacific Islander	12 (0 – 88)	15 (18)
% Any Non-White	16 (1 – 88)	18 (17)
% Hispanic Ethnicity	14 (1 – 38)	15 (15)
% Less than High School Education	24 (0 – 64)	23 (17)
% Medicare Insurance	18 (1 – 44)	17 (9)
% Admitted from Acute Hospital	82 (15 – 98)	71 (29)
***Comorbidities****(as*% *of all facility residents)*		
% Diabetes	27 (11 – 59)	31 (13)
% Skin Lesions	72 (4 – 100)	67 (23)
% Fecal Incontinence	44 (5 – 91)	43 (23)
% Indwelling Devices	2 (0 – 46)	12 (22)
% History of MRSA	11 (0 – 69)	13 (14)
***Functional Status****(average score among all facility residents)*		
Average Activities of Daily Living Score	19.82 (10.77 – 26.90)	19.74 (3.60)
Average Resource Utilization Group Score	0.92 (0.81 – 1.43)	1.0 (0.2)
Average Social Engagement Score	2 (0 – 4)	2 (1)

**Table 2:** MRSA Prevalence and Transmission Risk for 26 Nursing Homes

	**No. Beds**	**MRSA Admission Prevalence**^**a**^	**MRSA Point Prevalence**	**Estimated MRSA Transmission Risk**^**b**^
NH1	124	3% (3)	8% (8)	5%
NH2	59	4% (2)	22% (22)	19%
NH3	145	8% (8)	30% (30)	24%
NH4	208	9% (9)	19% (19)	11%
NH5	137	10% (5)	25% (17)	16%
NH6	24	11% (11)	7% (7)	0%
NH7	198	12% (12)	22% (22)	11%
NH8	80	13% (13)	25% (25)	14%
NH9	99	14% (7)	27% (27)	15%
NH10	99	16% (16)	31% (31)	18%
NH11	99	16% (16)	37% (37)	25%
NH12	98	16% (16)	39% (29)	27%
NH13	255	20% (20)	42% (42)	28%
NH14	99	21% (21)	16% (16)	0%
NH15	145	22% (11)	34% (34)	15%
NH16	138	22% (22)	30% (30)	10%
NH17	182	25% (25)	39% (39)	19%
NH18	99	29% (29)	44% (44)	21%
NH19	143	31% (31)	52% (52)	30%
NH20	46	-----	0% (0)	n/a
NH21	124	-----	2% (1)	n/a
NH22	45	-----	10% (4)	n/a
NH23	41	-----	16% (4)	n/a
NH24	99	-----	26% (13)	n/a
NH25	30	-----	27% (8)	n/a
NH26	46	-----	28% (10)	n/a
Mean (SD)	110 (58)	16% (8)	25% (13)	16% (8)
Median (range)	99 (24–255)	16% (3–31)	26% (0–52)	15% (0–30)

^a^ Admission prevalence swabs were not collected for nursing homes 20 through 26 due to small facility size and minimal resident turnover.

^b^ Estimated transmission risk was calculated as the absolute difference (MRSA point – admission prevalence) divided by the number of at risk patients per 100 admitted. For example, for NH3, transmission risk = (30% - 8%) / (100–8) = 24%.

**Table 3:** Bivariate Analysis of Factors Associated with Nursing Home MRSA Point Prevalence and MRSA Transmission Risk

**Variable**	**Absolute %Change MRSA Point Prevalence per 10% change in variable**	**p-value**	**Absolute %Change MRSA Transmission per 10% change in variable**	**p-value**
**Nursing Home Characteristic**				
Annual Admissions (per100 admissions)	1.02	0.09	0.6	0.24
Annual Resident Turnover	−5.9	<0.001	−2.7	0.06
Average Direct Care Nursing Staff Hours (per resident day)	1.8	0.92	−0.2	0.99
High MRSA Admission Prevalence^a^	14.3	<0.001	5.6	0.18
***Demographics****(as*% *of all facility residents)*				
Age under 65	−1.4	0.14	0.7	0.48
Age over 85	0.4	0.76	−1.9	0.09
Male Gender	−1.4	0.52	2.9	0.16
Hispanic Ethnicity	2.9	0.21	3.1	0.05
Non-White Race	1.5	0.33	1.6	0.11
Education less than High School	2.6	0.1	2.3	0.03
Admitted from Acute Hospital	2.4	0.006	0.4	0.71
***Comorbidities****(as*% *of all facility residents)*				
Diabetes	6.9	<0.001	3.6	0.01
Skin Lesions	2.2	0.05	−1.0	0.41
Fecal Incontinence	3.5	0.001	2.2	0.03
Indwelling Devices	2.0	0.09	1.9	0.14
History of MRSA	3.2	0.10	2.8	0.38
***Functional Status***				
High Resource Utilization Group^a^	4.5	0.25	1.2	0.76
% Residents with High Social Engagement Score	−2.1	0.19	−2.0	0.06

^a^MRSA admission prevalence and Resource utilization group (RUGS) score were dichotomized into high and low groups around median values.

Similar variables were found to be associated (p<0.1) in bivariate models predicting MRSA transmission risk, including resident turnover rate, the percent of residents >85, and percent of residents with diabetes or fecal incontinence. MRSA transmission risk was also associated with the percent of residents who were Hispanic, or had less than a high school education. Facility social engagement scores were negatively associated with MRSA transmission risk. Results for continuous variables are reported per 10% increase; for example, a 10% absolute increase in the resident turnover rate was associated with a 2.7% absolute decrease in MRSA transmission risk.

In multivariate models (Table 4), higher MRSA point prevalence was associated with higher MRSA admission prevalence and a higher percent of residents with indwelling devices. In this model, the percent of residents with indwelling devices and with fecal incontinence were interchangeable. MRSA transmission risk was associated with the percent of residents with diabetes and was negatively associated with social engagement level among residents. In our transmission model, the percent of residents with diabetes and with less than a high school education were interchangeable.

**Table 4:** Multivariate Linear Regression Analysis of Factors Associated with Nursing Home MRSA Point Prevalence and MRSA Transmission Risk

**Outcome: Facility MRSA Point Prevalence**		
**Variable**	**Absolute Change in MRSA Point Prevalence per absolute increase of 10% in variable(95% CI)**	**p-value**
High MRSA Admission Prevalence^a^	13.0 (4.3, 21.7)	0.005
% Residents with Indwelling Device^b^	1.8 (0.1,3.5)	0.04
**Outcome: Facility MRSA Transmission**		
**Variable**	**Absolute Change in MRSA Transmission per absolute increase of 10% in variable(95% CI)**	**p-value**
% Residents with High Social Engagement^c^	−2.0 (−3.8,-0.2)	0.03
% Residents with Diabetes^d^	3.6 (1.1,6.0)	0.01

^a^ MRSA admission prevalence was dichotomized at the median value (15%).

^b^ The presence of an indwelling device was collinear with the proportion of residents with fecal incontinence.

^c^ High social engagement score was defined as greater than or equal to 3 out of 6.

^d^ In the transmission model, the percentage of residents with diabetes was collinear with the percent of residents with less than a high school education.

## Discussion

In comparison to hospitals, the burden and predictors of MRSA in nursing homes are not well understood despite several studies suggesting MRSA prevalence may be much greater in this setting than high risk acute care wards, including intensive care units [12-16]. Nursing homes have a large concentration of high risk patients due to older age, chronic illness, and requirement for sustained nursing care. Nevertheless, the paucity of studies on factors associated with high burden and transmission or prevention may explain why approaches to infection prevention of multi-drug resistant organisms remain non-standardized in nursing homes [7,27-29].

In a large regional survey of nursing homes, we found that MRSA prevalence varied widely, from 0 to over 50%. Unsurprisingly, importation levels were strongly associated with overall prevalence, but, in addition, nursing homes caring for residents with more medical devices had significantly higher MRSA levels. This finding may be directly related to the portals of entry that devices provide for pathogens [3,18,30,31], or it may be reflective of a higher degree of chronic illness in that facility which leads to greater vulnerability for acquisition. Devices have also been associated with MRSA acquisition in hospital-based studies [31,32]. Among nursing homes that admitted similar proportions of residents with MRSA, some nursing homes were able to maintain their overall MRSA burden at or near importation levels, while other nursing homes had overall burden estimates that greatly exceeded importation levels. This suggests that MRSA transmission might be occurring in the latter group and that facilities in the former group may be employing specific strategies to successfully prevent MRSA levels from rising beyond the importation level. Further research is needed to understand whether differences in MRSA burden vs. importation are driven by facility practices, such as infection control policies or environmental cleaning protocols.

Among collected variables, we found that nursing homes with a higher proportion of residents with diabetes had higher estimated MRSA transmission, again suggesting that comorbidities are a marker of vulnerability [33-36]. Surprisingly, a high degree of social engagement among residents was protective of MRSA transmission, suggesting that the level of health needed to engage in activities outweighed the risk of transmission due to social contact. This was reassuring since nursing homes have a responsibility to promote residents’ emotional and physical health through social interaction, and this often precludes the adoption of stringent infection control policies found in acute care settings, such as isolation or long-term use of contact precautions.

This study has several limitations. First, we estimated MRSA transmission risk based upon MRSA admission and point prevalence. Second, our prevalence estimates were based upon nasal swabs and not sampling of multiple body sites, and we did not use enrichment techniques for culturing MRSA. As a result, the MRSA prevalences reported here may be an under-estimate of the true burden. Second, we did not collect information on facility practices that may influence MRSA burden, including infection control and environmental practices and frequency of antibiotic use. We also did not collect demographic information on residents who refused to be swabbed; these residents may have been substantially different from participants. However, the refusal rate was less than 5%, suggesting that this small number of non-participants would need to be quite different from participants to change our results. Finally, this is an ecologic study of facility-level characteristics associated with MRSA carriage. More research is needed to understand how facility-level factors influence individual risk of MRSA in this setting. Nevertheless, since infection control and prevention policies are determined on the facility level, facility characteristics may be helpful in identifying nursing homes that should adopt more aggressive strategies (e.g. screening, decolonization, more frequent environmental cleaning) to reduce MRSA burden and transmission.

## Conclusions

In a large, diverse metropolitan county, we found that MRSA burden varied substantially and was associated with both importation and facility-level indicators of comorbidity. However, we also found that higher levels of social engagement among residents were protective of MRSA transmission, a reassuring finding for the national movement toward increasing the community and homelike environment in nursing homes [37]. Identifying characteristics of nursing homes at risk for high MRSA burden may allow facilities to tailor infection control policies and interventions to mitigate spread of MRSA and other pathogens.

## Competing interests

The authors declare that they have no competing interest.

## Authors’contributions

CRM completed all statistical analyses and drafted the manuscript. VQ and DK participated in data collection and entry and contributed to study design. MW assisted with data entry and analyses. GT and KE processed collected nasal swabs. EP, HM, MC, BYL, DBM and SSH contributed to study design, and SSH also helped to draft the manuscript. All authors read and approved the final manuscript.

## Pre-publication history

The pre-publication history for this paper can be accessed here:

http://www.biomedcentral.com/1471-2334/12/269/prepub
